# Why are policies to combat undernutrition not effective in Mozambique?

**DOI:** 10.1038/s41467-026-69040-9

**Published:** 2026-02-03

**Authors:** Ana Raquel Ernesto Manuel Gotine, Sancho Pedro Xavier

**Affiliations:** 1 Postgraduate Program in Public Health, University of São Paulo (USP), São Paulo, SP Brazil; 2 Faculty of Health Sciences, Lurio University, Nampula, Mozambique

**Keywords:** Paediatric research, Health policy

## Abstract

Child malnutrition is high in Mozambique despite multiple policies and programs to improve the situation. In this Comment, the authors discuss the reasons for the lack of progress.

## Teaser

Despite multiple policies, child undernutrition in Mozambique remains high due to poverty, weak health and food systems, limited community engagement, and poor multisectoral integration. Strengthening coordinated, community-centered governance is urgently needed to ensure effective implementation and sustainable impact.

## Why policies to combat undernutrition are not effective in Mozambique?

Even with the existence of multiple policies and programs aimed at combating undernutrition the levels of child undernutrition in Mozambique remain high. The United Nations Development Program (UNDP) indicates that the most recent figure released for Mozambique’s Human Development Index (HDI) is approximately 0.461, classified as low human development^1^. According to the Mozambique Demographic and Health Survey (MDHS 2022-2023), around 37 per cent of children under the age of five had chronic undernutrition^2^. Furthermore, 73% of children suffer from anemia and micronutrient deficiencies, particularly vitamin A deficiency, remain highly prevalent^2,3^.

The challenges remain considerable, as approximately 63% of children live in multidimensional poverty, a situation that reflects simultaneous deprivation in essential aspects of well-being such as health, nutrition, education, housing, and sanitation^4,5^. In addition, nutritional deficiencies, especially anaemia, in adolescent mothers and women of reproductive age are a critical determinant of child health, particularly in the first 1000 days, from conception to early childhood^6^.

A recent survey reported a high prevalence of anemia (52%) in women of this age^7^. Another factor is the high frequency of recurrent infectious and diarrheal diseases in the Mozambican context, which exacerbate children’s nutritional vulnerability^8^. This scenario, characterized by persistent poverty, limited access to quality health services, sanitation infrastructure, and recurrent food insecurity, reflects the multifaceted nature of undernutrition in Mozambique, which may help to explain why existing policies have not achieved the expected results^9,10^.

In many rural and peri-urban areas, families rely on monotonous diets with low nutritional diversity, while health and nutrition services remain under-resourced and unevenly distributed^9^. Together, these factors help explain why existing policies and programs have not achieved the expected results, as most interventions are centrally designed and often fail to incorporate the local cultural, social, and environmental realities that shape nutritional practices and access to food and care^8,11^.

In rural communities, although traditional food practices, taboos, and cultural influences may affect child feeding in some settings, these factors are increasingly considered in the design and delivery of nutrition interventions in Mozambique by the Ministry of Health and its partners^12^. For example, a study carried out in Cabo Delgado - Mozambique, reported that eggs, bananas, honey, among other foods, were considered “potentially harmful” for children <2 years old or for pregnant/lactating women because of local beliefs/traditions^10^. Furthermore, implementation is compromised by weak health and food systems, as many communities face a chronic lack of trained professionals, inadequate infrastructure and logistical barriers that hinder the timely distribution of nutritional supplements and therapeutic foods^13^.

Another important aspect is community participation; studies show that nutrition programs with strong community engagement are more likely to succeed, however, many initiatives in Mozambique remain vertical and with little active participation of communities in the design, implementation and evaluation. The empowerment of women, for example, is a decisive factor for the success of interventions, as they are primarily responsible for feeding children^8,14^. Community-based nutrition programs implemented in Mozambique, particularly those focusing on infant and young child feeding practices and maternal nutrition, have demonstrated positive impacts, including improvements in dietary diversity and reductions in child undernutrition indicators in specific regions. Examples include initiatives supported by the United States Agency for International Development (USAID) and Food for the Hungry^15^, World Vision Mozambique^16^ and World Food Program^17^.

In addition, nutritional interventions are rarely integrated with sustainable agriculture programs, since most rural families depend on subsistence farming but face difficulties in diversifying crops and improving the nutritional value of available food, encouraging local production of nutritious food can enhance the effectiveness of interventions and reduce dependence on external supplements^18^. Evidence from household studies in Nampula indicates that even where agricultural production exists, local diets remain low in diversity and not fully supplied by that production, reflecting constraints on consumption despite availability (household food production does not straightforwardly translate into local consumption)^19,20^. Severe food insecurity prevents families from prioritizing healthy eating practices, even if they are aware of the recommendations, families choose between minimal food for survival and other basic needs, making it difficult to adopt nutrition messages that require regular access to diversified foods^21,22^.

Weak intersectoral coordination and insufficient monitoring jeopardize the long-term impact assessment of nutritional interventions^11^. Many policies and programs remain focused on one-off, short-term activities, rather than adopting integrated strategies that combine food security, improving basic sanitation, empowering women and strengthening health services, this results in duplication of efforts, inefficient use of resources, and a significant reduction in overall impact. In addition, nutrition education, which should be a central component, is still conducted in an isolated and sporadic manner, without managing to promote lasting behavioral changes^11,23,24^.

In Mozambique, two interrelated challenges compromise the effectiveness of programs to combat undernutrition. First, inadequate follow-up mechanisms limit the monitoring of program implementation and the evaluation of their nutritional impact on beneficiary children. Second, program coverage and reach remain limited, as logistical and structural barriers hinder the consistent delivery of micronutrient supplementation and the inclusion of all eligible children^25,26^.

On the other hand, the lack of regular home visits, systematic records, and trained human resources prevents the early detection of interruptions or dropouts, reducing the overall effectiveness of the intervention^26^. Given financial and logistical constraints, large-scale implementation of home visits may not be feasible, however, integrating periodic household follow-up into existing maternal and child health programs and leveraging community health workers could provide a more sustainable and accessible approach to monitor adherence and improve program continuity in remote areas^26^. In addition, the evaluation of projects and programs usually focuses on process indicators (such as the number of supplements distributed or sessions held), without assessing concrete nutritional results or improvements in anthropometric indicators^27,28^.

The Mozambican socio-political context also poses significant challenges, such as the political instability (in northern of the country, particularly in Cabo Delgado and parts of Nampula and Niassa provinces, recurrent terrorist attacks by insurgent groups since 2017 intensifying from 2022 onward), affects the logistics of delivery and the functioning of health units, compromising the continuity of programs^29,30^. In addition, recurrent climatic disasters such as cyclones, floods, and prolonged droughts have become a major factor contributing to food insecurity in Mozambique. Over the past decade, extreme weather events like Cyclones Idai and Kenneth in 2019 and Cyclone Freddy 2023 have devastated agricultural production, destroyed infrastructure, and displaced millions of people. These events not only undermine household food availability but also disrupt essential health and nutrition services, reversing the nutritional progress achieved in recent years and increasing the vulnerability of children and women in affected communities^14^.

To reverse the current situation, the Mozambican government needs to adopt a multisectoral, community-centered approach that respects local realities, strengthens health systems and tackles the social and structural determinants of undernutrition. This includes not just distributing supplements or promoting campaigns such as the National Strategy for the Prevention and Control of Micronutrient Deficiencies and the National Food Fortification Program, but also investing in infrastructure, improving access to water and sanitation, promoting diversified and climate-resilient agriculture, and empowering women through education, social protection initiatives, and income-generating opportunities^17^.

However, there is an urgent need to prioritize effectiveness and real impact studies, with robust monitoring and evaluation, to continually adjust strategies according to the results observed. Nevertheless, in the current global context of declining financial aid and constrained funding for development programs, implementing such evidence-based approaches poses additional challenges, limited resources may restrict the ability to carry out comprehensive evaluations or to scale successful interventions. This reinforces the importance of optimizing existing funds through multisectoral collaboration, integration of monitoring systems into routine health services, and capacity building at both the central and community levels^17^.

Without this paradigm shift, the objectives of public policies will remain more aspirational than transformative, and undernutrition will continue to be one of the main barriers to human development in the country^1^. Evidence from Malawi, Ethiopia, and Brazil demonstrates that multisectoral, community-centered approaches can substantially reduce child undernutrition.

In Malawi, community nutrition programs integrating supplementation, health education, and home gardening improved dietary diversity and child growth outcomes^31^. National stunting prevalence in children under 5 years declined by 10 percentage points (47% in 2010 to 37% in 2016) (“National Statistical Office (NSO). Malawi demographic and health survey 2010. Zomba (Malawi) and Calverton (MD): NSO and ICF Macro; 2011”, “National Statistical Office (NSO). Malawi demographic and health survey 2015–2016. Zomba (Malawi) and Rockville (MD): NSO and ICF; 2017”), with quasi-experimental evidence confirming the effectiveness of these interventions in rural districts^32^. In Ethiopia, community-based initiatives combining health, education, and agricultural actions contributed to a reduction in stunting (48% in 2000 to 36% in 2019), representing a 25% decline over two decades^33^. In Brazil, the National School Feeding Program (PNAE) links school meals with local food production and active parental and community participation, improving both nutritional and educational outcomes^34^ and was associated with a marked reduction in stunting among children under 5 years (37% in 1974–75 to 7% in 2006–07)^35^.

In conclusion, the persistence of child undernutrition in Mozambique shows that one-off, disjointed solutions are not enough in the face of a structural and multifaceted problem. It is a challenge that requires more than distributing supplements or promoting isolated campaigns; it also demands improved access to health services, regular growth monitoring, and anemia screening to ensure early detection and effective intervention. It requires a political commitment to integrated, sustainable actions that are sensitive to the local context. A community-centered approach that brings together health, nutrition, sanitation, agriculture and women’s empowerment can transform policies into concrete and lasting results.
